# Editorial: Neuromechanics in Movement and Disease With Physiological and Pathophysiological Implications: From Fundamental Experiments to Bio-Inspired Technologies

**DOI:** 10.3389/fphys.2022.895968

**Published:** 2022-04-14

**Authors:** Ramona Ritzmann, Alessandro Del Vecchio, Stéphane Baudry, Nicolas Place, Albert Gollhofer, Marco Narici, Christoph Centner

**Affiliations:** ^1^ Department of Sport and Sport Science, University of Freiburg, Freiburg, Germany; ^2^ Department of AI in Biomedical Engineering, University of Erlangen-Nuernberg, Erlangen, Germany; ^3^ Laboratory of Applied Biololgy and Research Unit in Applied Neurophysiology (LABNeuro), Faculty of Motor Sciences, Université Libre de Bruxelles, Bruxelles, Belgium; ^4^ Institut des Sciences du Sport, University of Lausanne, Lausanne, Switzerland; ^5^ Neuromuscular Physiology Laboratory, Department of Biomedical Sciences, University of Padua, Padua, Italy

**Keywords:** sensory, muscle, tendon, computational neuroscience, simulation, robotic, brain

## Neuromechanics: The Why and the How

**FIGURE 1:**
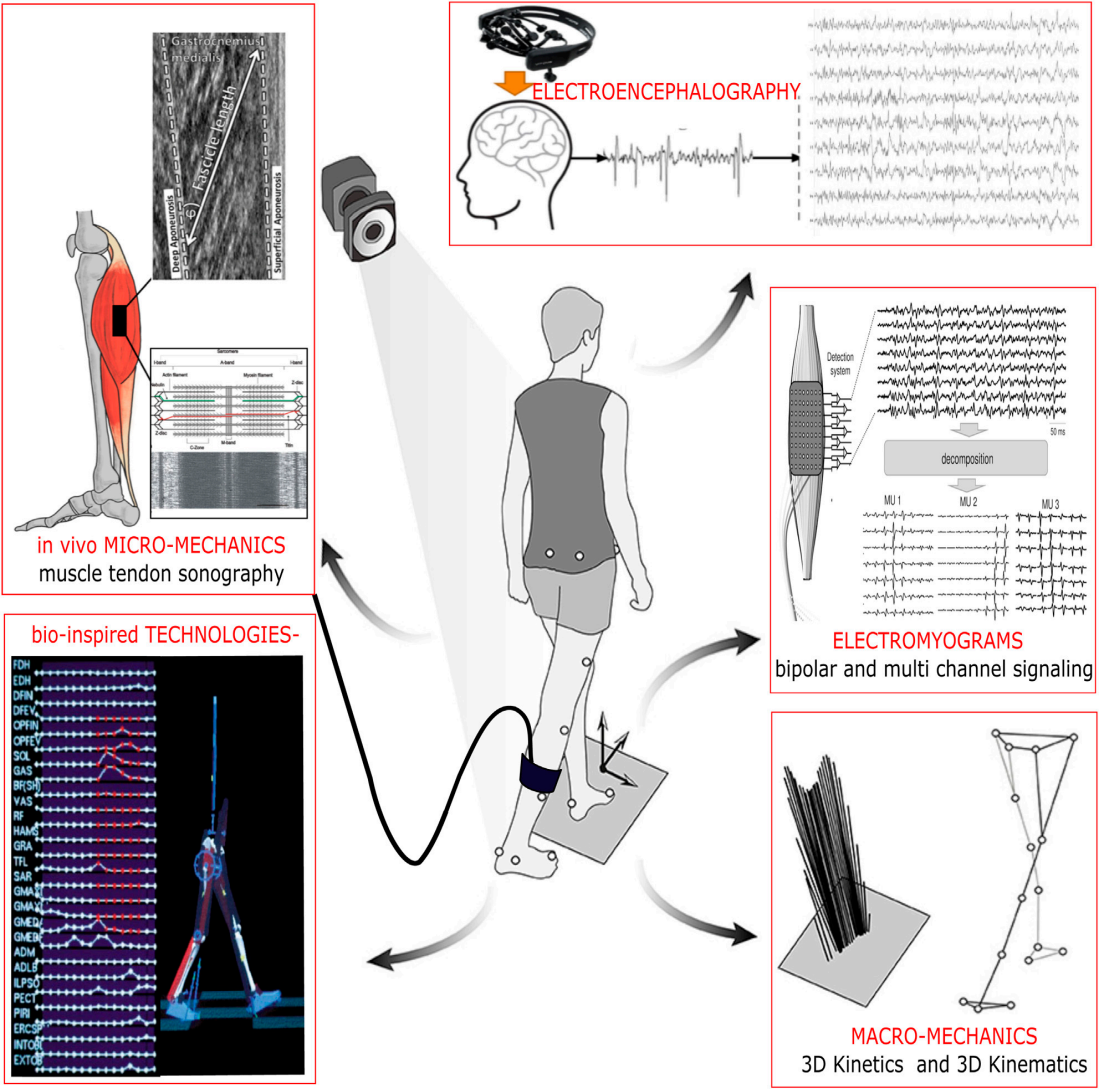
The neural control of human movement is characterized by the resulting micro- and micromechanics. Scientific evidence from holistic experiments is aiding in the development of new therapeutic approaches and bio-inspired technologies to improve motor control in healthy individuals and patients and trigger long-term adaptations.

Coordinated motor function in humans is characterized by a complex interplay between neuromuscular Kalc et al. and musculoskeletal elements Smart et al. Within the field of neuromechanics, neuroscience (e.g., assessment of neural control mechanisms *via* neuroimaging or neurophysiology for spinal and supraspinal areas), biomechanics (e.g., architectural muscle and tendon physiology, kinematics, kinetic characteristics), physiology (e.g., electromyography, *in vivo* bio-imagery) and technical approaches (e.g., computational neuroscience, humanoid robotics and bionic modelling) are combined to contribute to an holistic understanding of human movement Mohr et al.; Fauvet et al.; Ogasawara et al., through its underlying physiological processes and its adaptations to physical activity or chronic disuse Divjak et al. Transdisciplinary methodological techniques and integrative approaches exist to unravel paradigms of coordinated sensorimotor control, interventions and technologies to restore motor function, rehabilitation or movement optimization (Figure 1). These paradigms are used to predict the pattern of muscle activation Munoz-Martel et al. and the transmission of force *via* tendons to the skeleton Smart et al. They allow understanding how movement is anticipated Munoz-Martel et al., proactively generated and controlled in humans. Their clinical applications include easing health problems and designing and controlling bio-inspired robotic systems. Importantly, biological actuators are different from their mechatronic counterparts in terms of form and function Morasso; therefore coherence is achieved throughout progressive scientific evidence at the transdisciplinary conjunction. In this context, neuromechanics is not restricted to studying movement control Hofstetter et al., motor learning Morasso, in healthy individuals, but also helps to explain motor deficits in clinically relevant areas with reference to diseases and injuries.

## Physiological and Pathophysiological Implications

Examining the interplay between neural, and biomechanical and environmental dynamics serves as a unique approach for understanding the holistic framework of motor behaviors in both healthy and diseased individuals. Previous evidence indicates that biological and technical systems cannot be interpreted independently but must be integrated in a coherent context to allow to fully capture the complexity of movements (Ting et al., 2015). Several articles in this Research Topic tie in evidence about neuromuscular adaptations and deepen understanding about synergistic and agonistic muscle coordination with an impact on muscle-tendon interaction. For example, changes in micro- and macro-mechanical stress and strain Smart et al. were demonstrated to non-linearly affect monoarticular and complex motion of the entire body. Muscle pre-activation and synergies adopted to changes in movement anticipation Munoz-Martel et al., spinal excitability decreased with tissue flossing Kalc et al. and antagonistic co-activation Divjak et al. in the lower extremities was sensitive to the level of physical activity and life span in healthy humans. These neuromuscular modulations further took impact on myotendinous conjunction. Smart et al. and Stäudle et al. investigated muscle-tendon interaction and found that mechanical properties and energy management of the tendinous tissue is determined by neuronal innervation, contractile and architectural attributes of the attached muscle. In the transition between micro and macro mechanics and with an emphasis on more complex movements Hofstetter et al. showed that cervical spinal stiffness is segment and load dependent and Mohr et al. specified that upper body and pelvic rotation are sex-specific.

To contribute to the understanding of the pathogenesis of rapid eye movement sleep behavior disorder, Peng et al. analyzed electroencephalography (EEG) microstate characteristics with the aim of validating EEG microstate as an early-stage marker of this disorder. Their findings pointed towards abnormalities in resting-state EEG microstates and indicated that such neurophysiological analyses might complement current clinical concepts in the early detection of rapid eye movement sleep behavior disorder. Through the combined use of EEG, electromyographic (EMG) and kinematic measures, Fauvet et al. investigated the temporal dynamic of cortico-muscular coherence in post-stroke patients in order to investigate whether alterations of the functional coupling between brain and muscles contributes to impairments of motor function. The findings revealed that cortico-muscular coherence in antagonist muscles was higher for post-stroke patients compared to controls during the acceleration phase. The authors propose that this might reflect the loss of sensitivity of motor command occurring after stroke.

High-density EMG decomposition techniques allow an accurate and non-invasive evaluation of MU behavior in various populations and paradigms (Del Vecchio et al., 2020). In a study by Divjak et al., the authors compared the change in MU discharge patterns following 14-days of immobilization in young and older individuals. After analyzing isometric coactivations of the triceps surae and tibialis muscles, the authors demonstrated that changes in MU discharge rates and muscle coactivation patterns seem to be person-specific and dependent on the level of isometric loading. These results also highlighted that before immobilization, younger individuals demonstrated substantially higher inter-person variability in coactivation patterns, which have equalized following 14-days of immobilization.

## Bio-Inspired Technological Implications

In the light of the rapid progress in the field of advanced technological applications and robotics, bio-inspired assistive devices have been successfully designed and integrate physiological inputs to aid human movements Yang and Lee and compensate for impairments in human motor control. With the use of brain computer interfaces (BCIs), neural signals can be implemented to directly operate external devices with real-time feedback. In neurorehabilitation, signals from motor units convey important information about motor control which can be extracted and used to control robotic rehabilitation devices. Besides assessing motor unit behavior *via* invasive needle techniques (Adam et al., 1998), recent advances which use modern decomposition techniques (Del Vecchio et al., 2020) have been demonstrated as valid measures of the circuitries underlying the motor unit and thus motor behavior (Nordstrom et al., 1992; Heckman et al., 2005). From one side, motor unit and EMG properties during synergistic tasks such as locomotion or isometric contractions represent an interface with the neurorehabilitation intervention and supplementary characterization of the pathology, from another side it is possible to use the spared EMG activity after injury (e.g., spinal cord injury or stroke), for controlling assistive devices.

With this regard, BCIs (Collinger et al., 2013), electrical stimulation of the spinal cord (Wenger et al., 2016), and motor neuron interfaces (Farina et al., 2017) have shown the greatest potential in enabling the voluntary control of several degrees of freedom. Recently, Ting et al. (Ting et al., 2021) reported that it is possible to observe distinct patterns of EMG activity in a subject with complete spinal cord injury. Despite voluntary movement of the individual hand digits was not possible, the subject was able to control the activity of a few MUs that were unique for each finger. Future bio-inspired technologies can take advantage of these discharge patterns and novel finding being implemented in neuro-rehabilitation Munoz-Martel et al.

## Prospect

The present Research Topic aimed to overcome conventional boundaries of physiology, neuroscience and biomechanics to emphasize the interaction between the brain and muscles to produce adequate motor behavior in humans. The understanding of the underlying mechanisms coupled with bio-inspired application technologies will further empower us to revisit current approaches of robotic systems to produce human-like physical behavior or feasible applications in clinical or therapeutic environments. Given the high relevance of this topic, the Research Topic emphasizes fundamental practical applications useful for clinicians and exercise scientists.
